# Diagnosis and insight into the unique lung microbiota of pediatric pulmonary tuberculosis patients by bronchoalveolar lavage using metagenomic next-generation sequencing

**DOI:** 10.3389/fcimb.2024.1492881

**Published:** 2024-12-19

**Authors:** Haiyi Zhou, Yi Pei, Qifang Xie, Wenjie Nie, Xiaoyan Liu, Han Xia, Jie Jiang

**Affiliations:** ^1^ The Affiliated Changsha Central Hospital, Department of Center for Tuberculosis Diagnosis and Treatment, Hengyang Medical School, University of South China, Changsha, China; ^2^ Changsha Tuberculosis Technology Innovation Center of Children, Hengyang Medical School, University of South China, Changsha, Hunan, China; ^3^ Hunan Clinical Medical Technology Demonstration Base of Tuberculosis Diagnosis and Treatment, Hengyang Medical School, University of South China, Changsha, Hunan, China; ^4^ Department of Scientific Affairs, Hugobiotech Co., Ltd., Beijing, China; ^5^ Key Laboratory of Rare Pediatric Diseases, Ministry of Education, Hengyang Medical School, University of South China, Changsha, China

**Keywords:** pediatric pulmonary tuberculosis, bronchoalveolar lavage fluid, metagenomic next-generation sequencing, microecology, conventional microbial test

## Abstract

**Background:**

Although previous studies have reported the dysregulation of respiratory tract microbiota in infectious diseases, insufficient data exist regarding respiratory microbiota imbalances in the lower respiratory tracts of children with pulmonary tuberculosis (PTB). In this study, we assessed the value of mNGS in the pathogen diagnosis and microbiome analysis of PTB patients using bronchoalveolar lavage fluid (BALF) samples.

**Methods:**

A total of 64 participants, comprising 43 pediatric PTB and 21 pediatric pneumonia patients were recruited in the present study. BALF samples were collected from the above participants. Parallel comparisons between mNGS and conventional microbial test (CMT) pathogen detection were performed. Moreover, the diversity and structure of all 64 patients’ lung BALF microbiomes were explored using the mNGS data.

**Results:**

Comparing to the final clinical diagnosis, mNGS in BALF samples produced a sensitivity of 46.51%, which was lower than that of TB-PCR (55.00%) and Xpert (55.00%). The diagnostic efficacy of PTB can be highly enhanced by mNGS combined with TB-PCR (AUC=0.8140, *P*<0.0001). There were no significant differences in the diversity either between patients with TB and pneumonia. Positive mNGS pathogen results in pediatric PTB patients significantly affect the β-diversity of the pulmonary microbiota. In addition, significant taxonomic differences were found in BALF specimens from patients with PTB and pneumonia, both of which have unique bacterial compositions.

**Conclusions:**

mNGS is valuable in the etiological diagnosis of PTB, and can reveal pulmonary microecological characteristics. For pediatric PTB patients, the mNGS should be implemented early and complementary to CMTs.

## 1 Introduction

Tuberculosis (TB) remains a major public health problem for children and adolescents. In 2022, 7.5 million people were newly diagnosed with TB, the highest number since 1995, when WHO began global TB surveillance (Global Tuberculosis Report, 2023). Of these, 12% were children (Global Tuberculosis Report, 2023). However, data are suggesting that the true incidence of TB in children is threefold higher than the officially communicated figure (Dodd et al., 2014). Children are more likely than adults to have TB disease with low bacterial loads (ie, paucibacillary disease), with resulting culture yields as low as 25%–40% (Thomas, 2017).

Pulmonary TB (PTB) is the most common presentation but diagnosis can be challenging due to nonspecific signs and symptoms, paucibacillary disease, and difficulties in obtaining adequate samples for children (He et al., 2021; Mandal et al., 2017). The clinical presentation of pediatric PTB patients is similar to that of other infectious pneumonia caused by bacteria/viruses, and misdiagnosis as pneumonia can lead to wrong/delayed treatment and patient management. Traditional methods have limited diagnostic efficiency in current clinical practice. Metagenomic next-generation sequencing (mNGS) has emerged as a promising approach for the detection of common, rare, and emerging microorganisms (Chiu and Miller, 2019; Gu et al., 2019; Hogan et al., 2021). Without relying on traditional cultures or requiring specific amplification, mNGS is unbiased in its ability to sequence small amounts of host and microbial nucleic acids extracted from a variety of clinical specimens to detect and identify pathogens (Han et al., 2019; Miller and Chiu, 2021), providing advantages in infection diagnostics, pathogen detection, and clinical therapeutic guidance (Hogan et al., 2021; Lee et al., 2020; Tao et al., 2022).

Current strategies to remedy PTB depend heavily on long-term medication, possibly accompanied by a high risk of severe adverse drug reactions, which has a more pronounced impact on pediatric PTB patients (Haas and Belknap, 2018; Prasad et al., 2019; Turkova et al., 2022). Most of the studies indicate that a complex and dynamic interaction between host and *M. tuberculosis* contributes to TB pathogenesis, so in addition to canonical pathogen-directed strategies, host-directed therapy is a novel and promising approach to anti-TB treatment, and the host microbiota is considered a potential target for improving the clinical outcomes (Li et al., 2024; Roquilly et al., 2019). The application of culture-independent techniques to investigate the lung microbiota has changed our previous viewpoint that healthy lungs are sterile. Despite the rapid development of human microbiota research, the number of available studies on the lung microbiota in the context of PTB remains limited, especially in pediatric PTB patients (Hu et al., 2020). Most of them used sputum as an indicator for the microbiota of the lung and lower respiratory tract (Sala et al., 2020; Ticlla et al., 2021; Valdez-Palomares et al., 2021). However, sputum is easily contaminated by microbes residing in the upper respiratory tract during expectoration, leading to an inability to authentically reflect the profiles of the lung microbiota. To date, no studies have examined the microbiota using samples of bronchoalveolar lavage fluid (BALF) from pediatric PTB, which is closer to the real profile of the microbiota in pediatric lungs, due to the difficulty in obtaining BALF specimens from pediatric PTB patients.

In this study, we firstly investigated the diagnostic value of mNGS for PTB in children by collecting BALF samples from pediatric PTB and pneumonia (non-TB, NTB) patients for mNGS and compared its detection performance with conventional TB microbiological tests (CMTs). We further analyzed the differences in the microbiota profiles of the lower respiratory lungs of pediatric PTB and NTB patients to characterize the lung microbiota of pediatric PTB patients by the microbiota in BALF samples.

## 2 Material and methods

### 2.1 Ethics and study design

The study was performed in accordance with the declaration of Helsinki and was approved by the ethics committee of the Changsha Central Hospital (2022-S0200), and written informed consent was signed by all the children and their parents. A total of 43 pediatric PTB patients (PTB group) and 21 pneumonia patients (NTB group) from the Department of Student and Child Tuberculosis of Changsha Central Hospital Affiliated to Hengyang Medical College, University of South China between August 2021 and June 2023 were retrospectively enrolled in this study.

Patients in the PTB group were: (1) showing positive TB culturing, TB-PCR, or mNGS result, which represent the gold standard of PTB diagnosis according to the WHO guidelines; or (2) based on the comprehensive evaluation of clinical manifestations, auxiliary test results (including AFB and Xpert), and outcome assessment after TB drug administration (Lewinsohn et al., 2017; WHO Guidelines Approved by the Guidelines Review Committee, 2022).

Patients in the NTB group were: (1) Manifestations of fever, cough, expectoration, refusal to eat, lethargy, irritability, wheezing, dyspnea, and others; (2) The respiratory rate increased: the respiratory rate ≥ 60 times/min in patients less than 2 months of age; respiratory rate ≥ 50 times/min between 2 months and 1 year old; respiratory rate ≥ 40 times/min in patients of 1-5 years old; respiratory rate ≥ 30 times/min in patients over 5 years old; (3) On physical examination of pulmonary signs, mild dullness may be observed on percussion, and fine wet rales or crepitations may be heard; (4) Imaging of the lungs showed patchy exudation.

The exclusion criteria were: (1) ≤ 28 days or > 18 years of age; (2) Non-infectious factors, such as congenital heart disease, pulmonary edema, asthma, upper airway obstruction, or pulmonary cystic fibrosis; (3) Contraindications to fiberoptic bronchoscopy, such as patients with severe cardiopulmonary dysfunction and coagulation dysfunction; (4) Incomplete clinical data; (5) Undetermined prognosis and clinical outcomes.

The final clinical composite diagnosis was decided by two experienced clinical experts jointly according to clinical characteristics and the results of mNGS and CMTs. In cases of inconsistent diagnosis, a third expert would adjudicate.

### 2.2 Sample collection

BALF of all patients was collected according to the recommendations of the European Respiratory Society (de Blic et al., 2000). Sedation and topical anesthesia were administered, and age-appropriate pediatric flexible fiberoptic bronchoscopes were selected. More severely diseased regions in patients with diffuse lung disease or the right middle lobes were selected, based on radiological findings or evidence from bronchoscopy. Warm saline (1 mL/kg body weight, maximum 20 mL per fraction) was dripped into the selected lung lobes, and at least 40% of the fluid was recovered by mechanical suction using a pressure of approximately 50 to 100 mmHg. Permission was obtained from the patient’s parents, and written informed consent was obtained prior to BALF collection. Sputum, throat swabs, peripheral blood, and other sample types were also collected for CMTs.

### 2.3 DNA extraction and sequencing

200 μL BALF were used for DNA extraction. The DNA from each sample was extracted and purified using the QIAamp DNA Micro Kit (QIAGEN, Hilden, Germany) following the manufacturer’s instructions. The concentration and quality of extraction were tested through Qubit 3.0 Fluoremeter (Invitrogen, Q33216) and agarose gel electrophoresis (Major Science, UVC1-1100). DNA libraries were constructed using QIAseq Ultralow Input Library Kit (QIAGEN, Hilden, Germany). Library quality control was performed by Qubit 3.0 Fluoremeter (Invitrogen, Q33216) and Agilent 2100 Bioanalyzer (Agilent Technologies, Palo Alto, USA). Subsequently, the inspected libraries were sequenced on an Illumina Nextseq 550 (Illumina, San Diego, USA) using the SE75bp sequencing strategy, with the instrument targeting 5-20 million reads per library.

### 2.4 Bioinformatics analysis

After obtaining the sequencing data, high quality data was generated by filtering out connectors, low quality, low complexity and shorter sequences. Next human-derived sequences matching to the human reference database (hg38) were removed by using SNAP software. The remaining data were then aligned to the microbial genome database using Burrow-Wheeler Alignment. This database contains a large collection of microbial genomes from NCBI containing more than 30,000 microorganisms, including 17,748 species of bacteria, 11,058 species of viruses, 1,134 species of fungi, and 308 species of parasites. *Mycobacterium tuberculosis complex (MTBC)* was considered positive when at least 1 read was mapped to either the species or genus level due to the difficulty of DNA extraction and low possibility for contamination (Miao et al., 2018; Simner et al., 2018). The microbial composition of the samples was determined. Finally, the above remaining data were also devoted to perform microbiota taxonomic diversity and the relative abundance calculation, respectively, using kraken2 and bracken softwares.

### 2.5 Statistical analysis

We converted read abundance to percentages based on the total number of high-quality mapped sequences for each sample at the species and genus levels. These normalized data were used for all subsequent statistical analyses. All statistical analyses were performed using R (version 4.2.1, R Foundation for Statistical Computing, Vienna, Austria) software, SPSS (version 27.0, IBM Corporation, Armonk), and GraphPad Prism (version 10.1.2, GraphPad Software Inc., San Diego). The area under the curve (AUC) of the receiver operating characteristic curve (ROC) was calculated to assess the performance in the prediction of disease classification. Qualitative data were compared between groups using the Wilcoxon rank-sum test or an independent t-test, and quantitative data were determined via crosstabs with the chi-square test. The Wilcoxon rank-sum test was used to compare alpha diversity measures. NMDS (non-metric multidimensional scaling) was used to compare beta diversity measures. ANOSIM was performed to test for the statistical significance of beta diversity. LEfSe analysis and ALDEx2 were used to estimate microbiota with differential abundance among the groups. A two-sided *P* value of less than 0.05 was considered statistically significant.

## 3 Results

### 3.1 Patient demographics

A total of 64 pediatric participants, comprising 43 in the PTB group (25 males, 18 females) and 21 in the NTB group (16 males, 5 females) were recruited in the study based on their clinical symptoms, laboratory tests, and imaging. The characteristics of the enrolled participants are shown in **Table 1**. No statistically significant differences were observed in terms of age and gender between the PTB group and the NTB group. There was a history of previous tuberculosis exposure in 44.19% of PTB patients. The rate of weight loss in the PTB group reached 44.19% and the length of hospitalization was significantly higher compared to the NTB group (*P*<0.05). After anti-tuberculosis treatment for the PTB and anti-infection treatment for the NTB, patients in both groups were discharged with improvement (**Table 1**).

**Table 1:** Clinical characteristics of patients enrolled in this study.

Characteristics	PTB (n=43)	NTB (n=21)	*P*-value
**Age years, median (IQR)**	11.00 (9.000)	6.000 (9.000)	0.3088
<2 years, n (%)	3 (6.976%)	2 (9.524%)	0.6006
2–5 years, n (%)	8 (18.60%)	5 (23.81%)	
6–10 years, n (%)	8 (18.60%)	6 (28.57%)	
11–17 years, n (%)	24 (55.81%)	8 (38.10%)	
Gender			
Male, n (%)	25 (58.14%)	16 (76.19%)	0.1790
Female, n (%)	18 (41.86%)	5 (23.81%)	
Clinical presentations			
Fever, n (%)	12 (27.91%)	9 (42.86%)	0.2659
Cough, n (%)	28 (65.12%)	13 (61.90%)	>0.9999
Night sweat, n (%)	3 (6.980%)	0 (0.000%)	0.5449
Loss of weight, n (%)	19 (44.19%)	1 (4.760%)	**0.0013**
**Tuberculosis exposure history, n (%)**	19 (44.19%)	1 (4.760%)	**0.0013**
**Hospital stays (day), Median (IQR)**	13.00 (9.000)	10.50 (7.500)	**0.0423**
**Improvement and discharge, n (%)**	43 (100.00%)	21 (100.00%)	>0.9999

Significance is indicated by bold marking when P<0.05.

### 3.2 TB−detection performance comparison between mNGS and clinical tests

**Figure 1:**
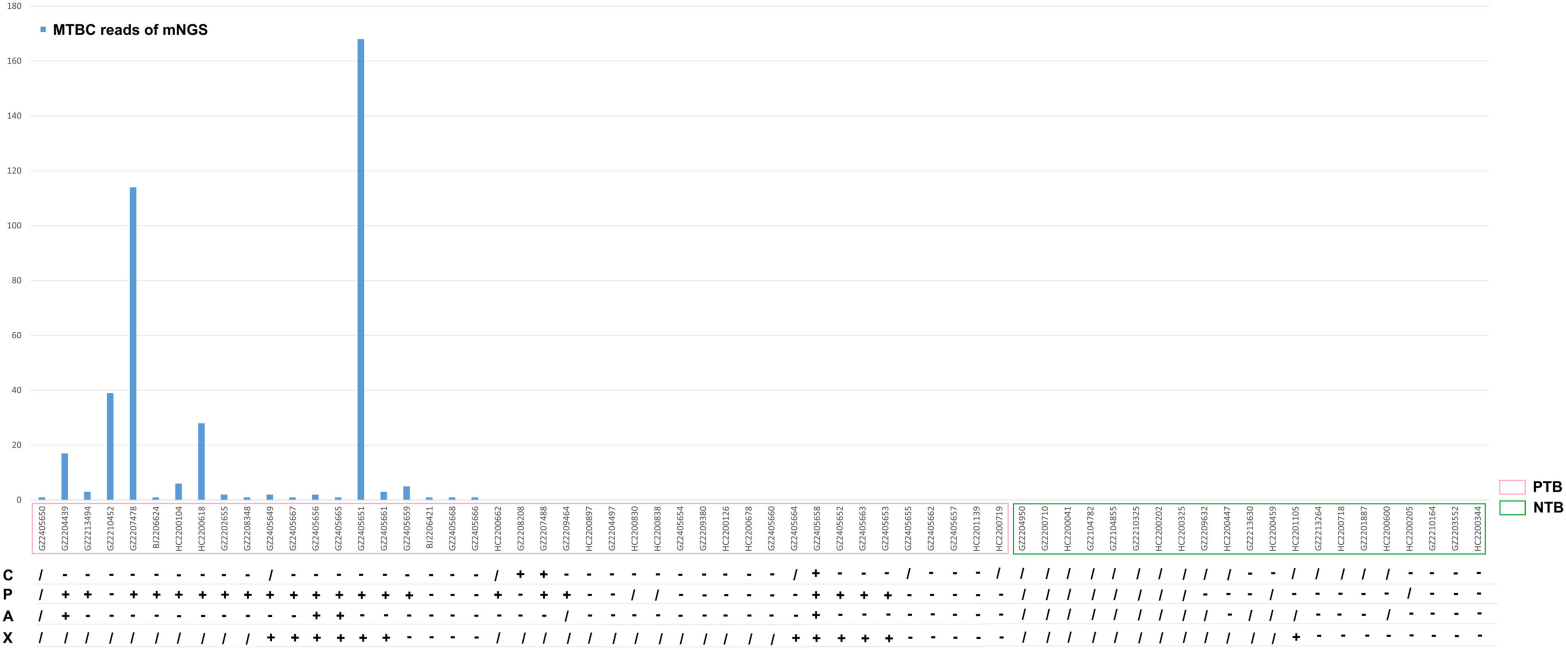
Culture, TB-PCR, AFB, X-pert results and MTBC Reads of mNGS for all subjects. C, culture; P, TB-PCR; A, AFB; X, X-pert;/, NA, unavailable; +, positive; -. negative.

In this study, multiple clinical tests including culture, TB-PCR, AFB, and Xpert, as well as mNGS using BALF samples were performed for TB detection (**Figure 1**). Due to the nature of the retrospective clinical study, the results of culture, TB-PCR, AFB, and Xpert were undetermined in 20, 14, 15, and 35 patients, respectively (**Table 2**; **Figure 1**). In the PTB group (n=43), over 67.44% of patients (29/43) were defined as test-defined TB, who had positive TB culturing or TB-PCR or mNGS-positive (**Table 2**; **Supplementary Figure 1**). While the remaining 14 PTB patients (32.56%) were diagnosed based on comprehensive clinical evidence (see Methods). In comparison, the culture and AFB assay demonstrated the lowest positive detection rate (7.89%, 9.76%) among all clinical tests (mNGS 46.51%, TB-PCR 55.00%, and Xpert 55.00%). In addition, 1 individual in the NTB group had a false-positive Xpert result (**Tables 2**, **3**).

**Table 2:** PTB-detection performance comparison between mNGS and clinical tests.

	PTB (n=43)	NTB (n=21)	*P*-value
**mNGS**			**<0.0001**
+	20	0	
−	23	21	
NA	0	0	
**Culture**			>0.9999
+	3	0	
−	35	6	
NA	5	15	
**TB-PCR**			**0.0013**
+	22	0	
−	18	10	
NA	3	11	
**AFB**			>0.9999
+	4	0	
−	37	8	
NA	2	13	
**Xpert**			**0.0432**
+	11	1	
−	9	8	
NA	23	12	

NA, unavailable. Significance is indicated by bold marking when P<0.05.

**Table 3:** Diagnostic ability of different test modalities for PTB patients.

	Sensitivity	Specificity	PPV	NPV	ROC AUC	ROC *P*-value
**mNGS**	46.51% (0.3251-0.6108)	100% (0.8454-1.000)	100% (0.8389-1.000)	47.73% (0.3375-0.6206)	0.7326 (0.6135-0.8516)	**0.0027**
**TB-PCR**	55.00% (0.3983-0.6929)	100% (0.7225-1.000)	100% (0.8513-1.000)	35.71% (0.2071-0.5417)	0.7750 (0.6462-0.9038)	**0.0076**
**Xpert**	55.00% (0.3421-0.7418)	88.89% (0.5650-0.9943)	91.67% (0.6461-0.9957)	47.06% (0.2617-0.6904)	0.7194 (0.5264-0.9125)	0.0626
Combined detection*	62.79% (0.4786-0.7562)	100% (0.8454-1.000)	100% (0.8754-1.000)	56.76% (0.4091-0.7133)	0.8140 (0.7124-0.9155)	**<0.0001**

*, mNGS combined TB-PCR detection; NPV, negative predictive value; PPV, positive predictive value.

The data utilized in this study did not differentiate between training and test sets, which may lead to limited generalizability of the statistical findings. Significance is indicated by bold marking when P<0.05.

Next, we evaluated the detection performance of three methods, including mNGS, TB-PCR, Xpert, and combined method of mNGS and TB-PCR. As shown in **Table 3**, the AUC of mNGS/TB-PCR to distinguish PTB and NTB were 0.7326/0.7750 (sensitivity: 46.51%/55.00%, specificity: 100%/100%) (*P*<0.05), which were significantly higher than that of Xpert (*P*>0.05). The combined method of mNGS and TB-PCR further increased the AUC to 0.8140 (sensitivity: 62.79%, specificity: 100%) (*P*<0.0001) (**Table 3**).

### 3.3 Biodiversity between pediatric PTB and NTB group

**Figure 2:**
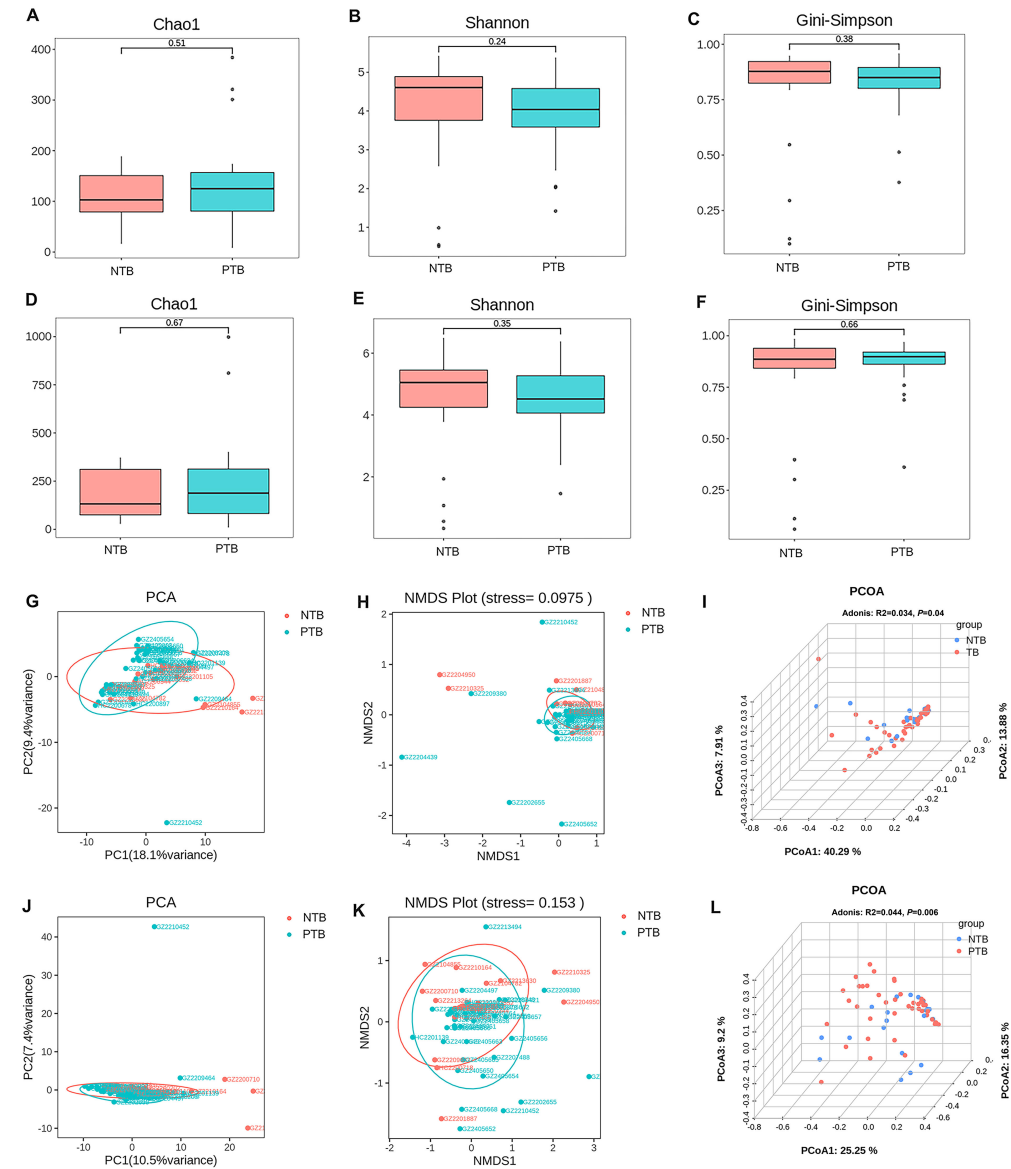
Comparison of α-diversity and β-diversity of the total microbial community in the lungs of pediatric patients with PTB and NTB. **(A-C)** α-diversity indexes of the total microbial community of the lungs between the two groups at genus level; **(D-F)** α-diversity indexes of the total microbial community of the lungs between the two groups at species level; **(G-I)** β-diversity indexes of the total microbial community of the lungs between the two groups at genus level; **(J-L)** β-diversity indexes of the total microbial community of the lungs between the two groups at species level.

In this study, 3 common indices, Chao1, Shannon, and Gini-Simpson, were selected to compare the α-Diversity of lung microbial communities of pediatric patients in the PTB and NTB groups. The Chao1 index was used to measure the number of species in the community, the Shannon index was positively correlated with richness and evenness, and the Gini-Simpson index was negatively correlated with richness and evenness. The Wilcoxon rank-sum test was performed to determine the significance of the differences in the index values between the two groups. At both genus and species levels for all microorganisms, the three α-diversity indices were not statistically significant between the two groups (*P*>0.05) (**Figures 2A-F**). This suggests that the species richness and homogeneity of the total microbial community in the lungs of PTB patients is similar to that of NTB patients.

Further PCA, NMDS, and PCOA analyses based on the Bray-Curtis distance matrix were used to compare β-diversity between groups, and the statistical significance of the PCOA results was analyzed for significance using Adonis. β-diversity is a measure of differences in overall microbiota composition between groups. For both genus-level and species-level microbiota, PCA and NMDS analyses showed no significant separation of the PTB and NTB groups (**Figures 2G, H, J, K**). Adonis analysis yielded *P*= 0.04 and R^2^ = 0.034 at the genus level, and *P*= 0.006 and R^2^ = 0.044 at the species level, suggesting significant differences between the structural composition of the total microbial community of the lungs between children PTB and NTB patients (**Figures 2I, L**).

### 3.4 Analysis of differences in the microbiota community between pediatric PTB and NTB

**Figure 3:**
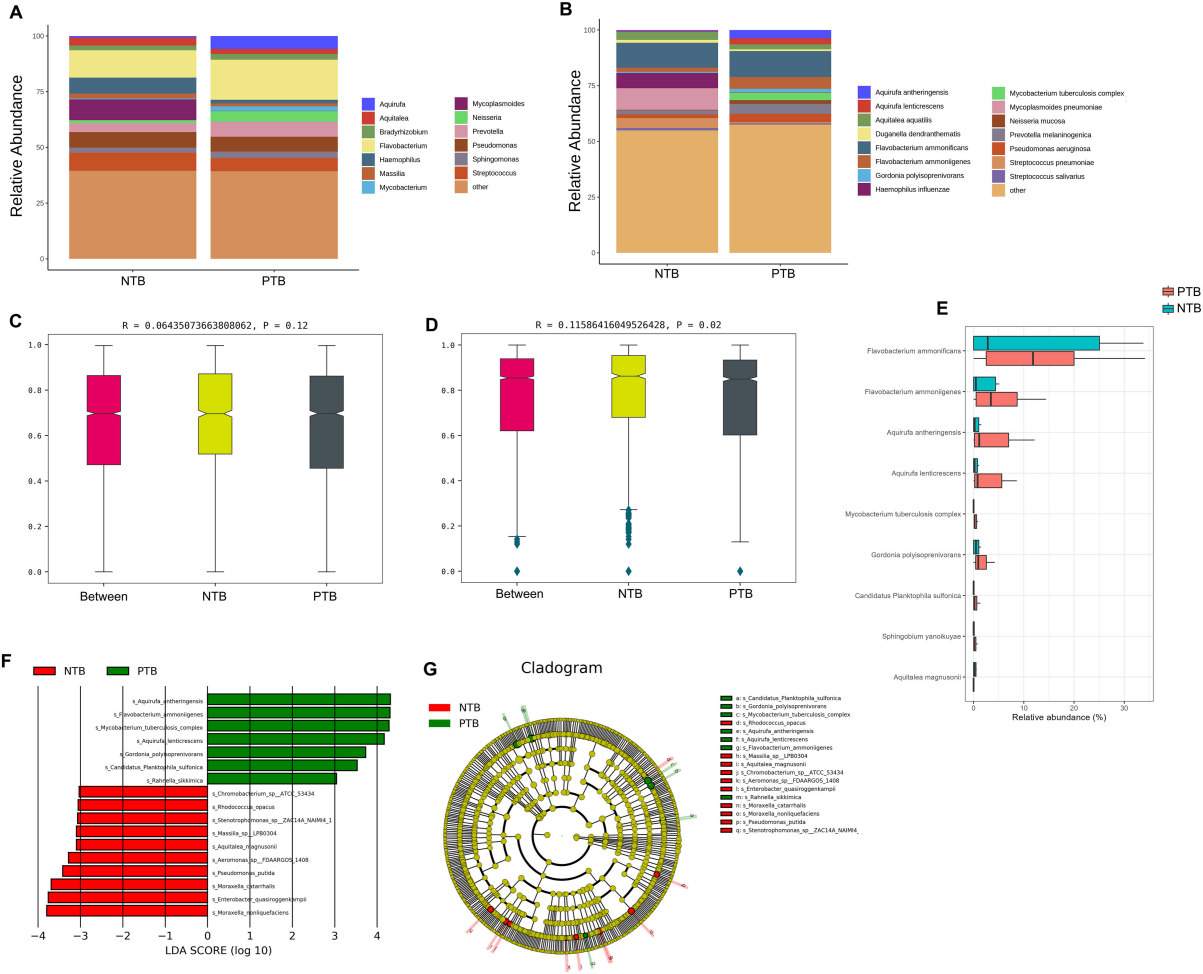
The difference in the distribution of pathogens between PTB and NTB patients. **(A, B)** The relative abundance of microorganisms between the two groups at the genus and species level; **(C, D)** Anosim analysis between the two groups at the genus and species level; **(E)** ALDEx2 analysis was performed to compare microbiome data differences after central log-ratio transformation of data; **(F, G)** LEfSe analysis was performed to identify differentially abundant taxa, which are highlighted on the phylogenetic tree in cladogram format **(F)** and for which the linear discriminant analysis scores are shown **(G)**.

The average relative abundance of *MTBC* was 3.40% in the PTB group and 0% in the NTB group (*P*<0.05) (**Supplementary Tables 1, 2**). The top 10 microbiotas with the largest relative abundance in each sample were selected as the dominant flora based on the genus and species level relative abundance table. **Figures 3A, B** shows that there were differences in the composition of the top 10 species in relative abundance of PTB and NTB, and which was similar in genus-level. Anosim analysis is a nonparametric test that is used to test whether the differences between groups are significantly greater than the differences within groups to determine whether the subgroups are meaningful. Further results of Anosim analysis confirmed statistical significance between the pediatric PTB and NTB groups at the species level (*P*<0.05) (**Figures 3C, D**).

The results of the diversity analysis showed that there were some differences in lung microbiota between pediatric PTB and NTB groups. ALDEx2 and LEfSe analysis was used to further compare the microbiota, with significantly different abundances among different groups to identify potential microbial biomarkers. ALDEx2 showed 9 differential strains between the two groups, with *Flavobacterium ammonificans*, *Flavobacterium ammoniigenes*, *Aquirufa antheringensis*, *Aquirufa lenticrescens*, and *Mycobacterium tuberculosis complex* being enriched (*P*<0.05) (**Figure 3E**; **Supplementary Table 2**). The LEfSe results showed that 17 bacteria with significant abundance differences were identified at the species level (*P*<0.05) (**Figures 3F, G**; **Supplementary Table 2**). *Aquirufa antheringensis*, *Flavobacterium ammoniigenes*, *Mycobacterium tuberculosis complex*, *Aquirufa lenticrescens*, and *Gordonia polyisoprenivorans* were enriched in pediatric PTB patients. *Moraxella nonliquefaciens Enterobacter quasiroggenkampii*, *Moraxella_nonliquefaciens*, *Pseudomonas putida*, and *Aeromonas sp FDAARGOS 1408* were enriched in pediatric NTB patients (**Figures 3F, G**).

### 3.5 Respiratory microbiota associated with TB based on different diagnostic standards

**Figure 4:**
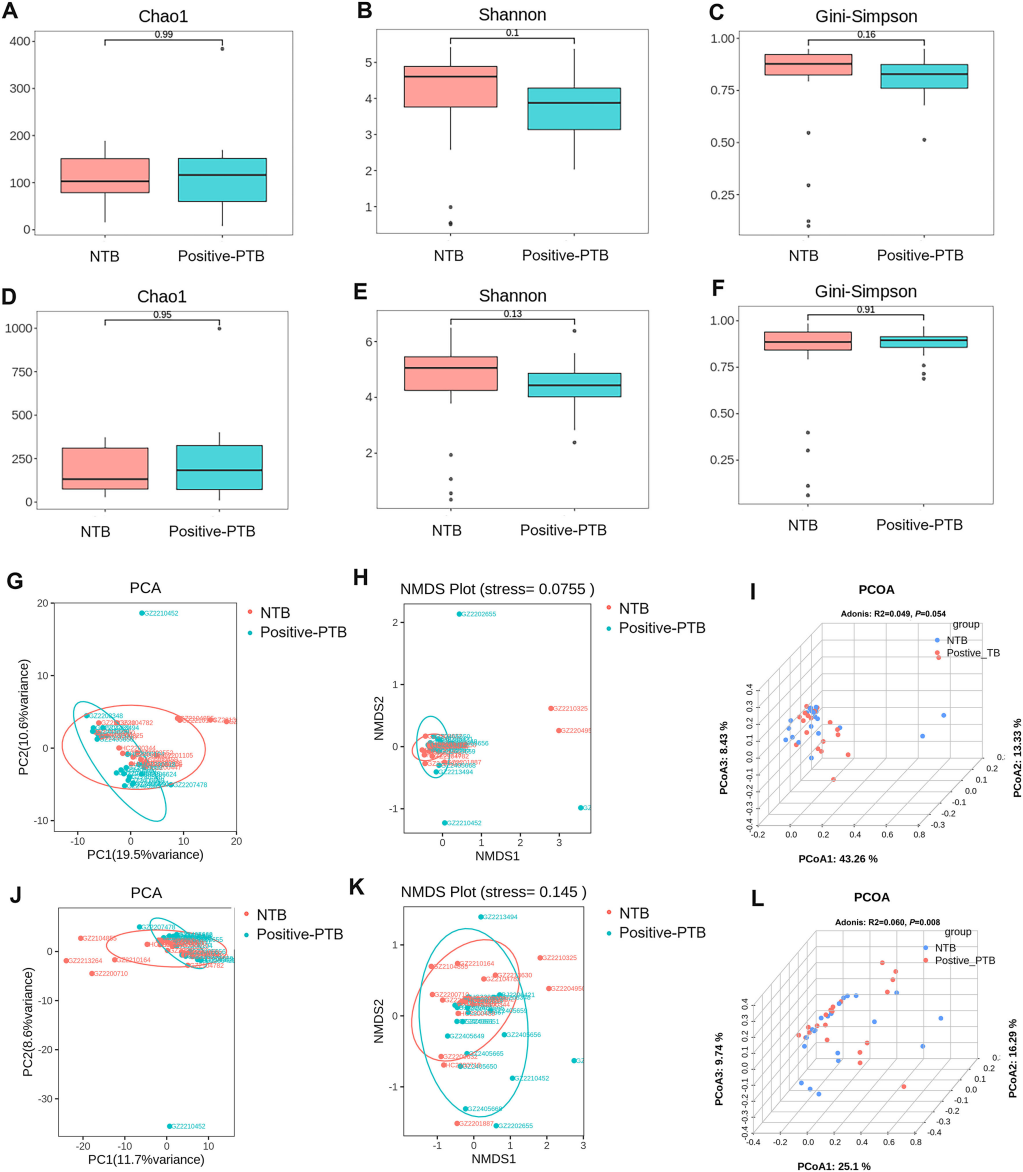
Comparison of α-diversity and β-diversity of the total microbial community in the lungs of pediatric patients with Positive-PTB and NTB. **(A-C)** α-diversity indexes of the total microbial community of the lungs between the two groups at genus level; **(D-F)** α-diversity indexes of the total microbial community of the lungs between the two groups at species level; **(G-I)** β-diversity indexes of the total microbial community of the lungs between the two groups at genus level; **(J-L)** β-diversity indexes of the total microbial community of the lungs between the two groups at species level.

We attempt to describe the characteristics of and changes in lung microbiota from different diagnostic standards. According to mNGS results, 20 pediatric PTB patients were further divided into positive-mNGS TB group (Positive-PTB). We first calculated the Chao1, Shannon and Gini-Simpson index to estimate the α-diversity of the microbial communities among the NTB and Positive-PTB groups. The results showed that although the Shannon index of the Positive-PTB group showed a decreasing tendency, there was no significant difference in α-diversity between the two groups at either species or genus level (*P*>0.05) (**Figures 4A-F**). It was shown that the species richness and homogeneity of the total microbial community in the lungs of pediatric Positive-PTB patients were similar to those of pediatric NTB patients.

β-Diversity was analyzed based on PCA, NMDS and PCOA analysis, and Adonis was used to determine the statistical significance of the PCOA results. For both genus-level and species-level microbiota, PCA and NMDS analyses showed no significant separation of the Positive-PTB and NTB groups (**Figures 4G, H, J, K**). Adonis analysis yielded *P*= 0.054 and R^2^ = 0.049 at the genus level, and *P*= 0.008 and R^2^ = 0.060 at the species level, suggesting significant differences in the species-level composition of the total lung microbial community pediatric Positive-PTB and NTB patients (**Figures 4I, L**).

**Figure 5:**
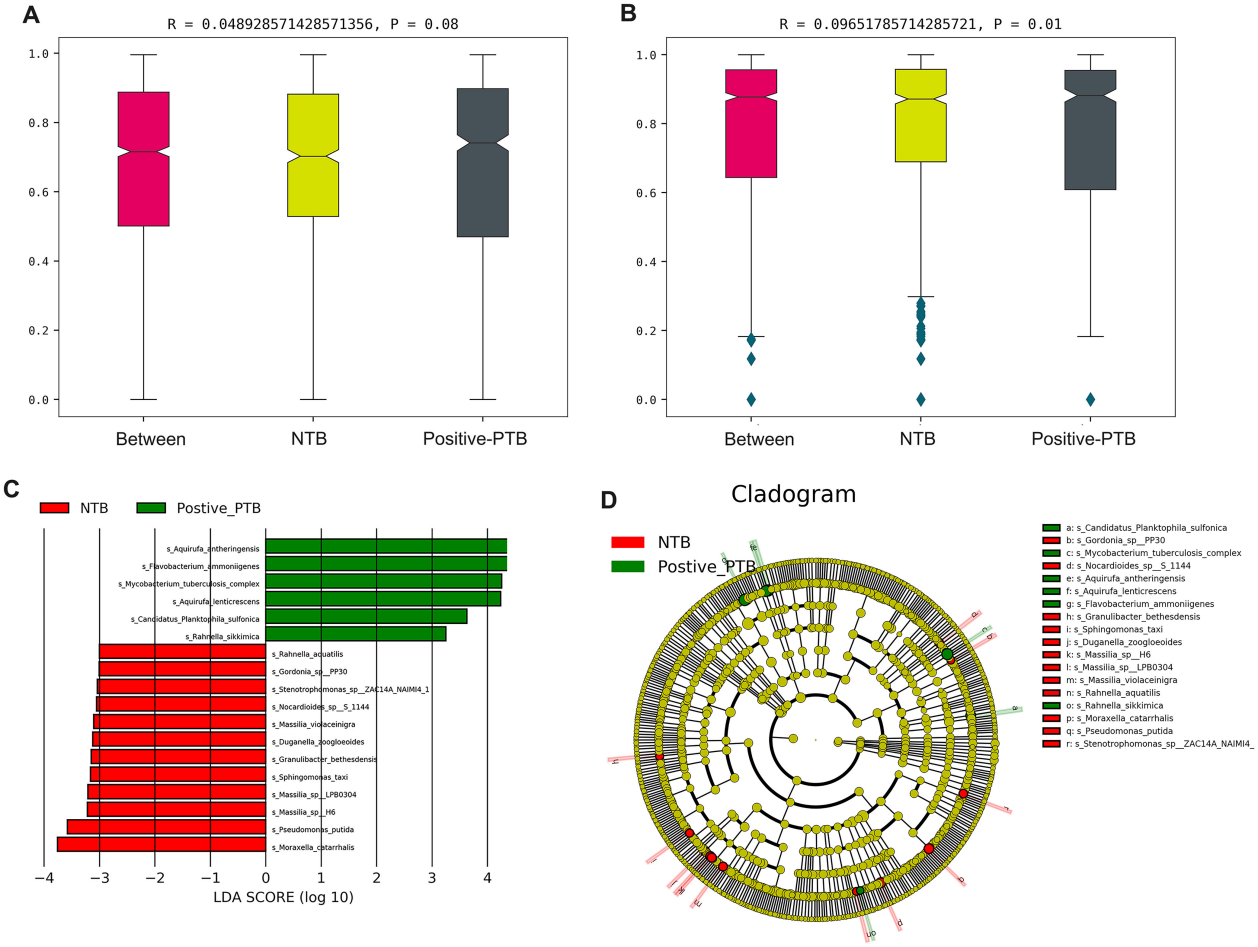
The difference in the distribution of pathogens between Positive-PTB and NTB patients. **(A, B)** Anosim analysis between the two groups at the genus and species level; **(C, D)** LEfSe analysis was performed to identify differentially abundant taxa, which are highlighted on the phylogenetic tree in cladogram format.

Further results of the Anosim analysis confirmed the statistical significance between the paediatric positive PTB and NTB groups at the species level (P<0.05), although there was no difference at the genus level (P>0.05) (**Figures 5A, B**). To identify the specific taxa responsible for the collective differences in community composition, we compared the relative abundance of prominent taxa in Positive-PTB patients and the NTB using a LEfSe method. In total, we detected 18 groups of bacteria with significantly different abundances. At the species level, *Aquirufa antheringensis, Flavobacterium ammoniigenes*, *Mycobacterium tuberculosis complex*, *Aquirufa lenticrescens*, *Candidatus Planktophila sulfonica*, and *Rahmella sikkimica* species were enriched in Positive-PTB, while *Moraxella catarrhalis*, *Pseudomonas putida*, *Massilia sp H6*, *Massilia sp LPB0304*, and *Sphingomonas taxi* species were enriched in NTB (**Figures 5C, D**).

## 4 Discussion

The value of BALF as being an excellent specimen for the etiologic diagnosis of tuberculosis has been confirmed before (Shi et al., 2020). In this study, BALF specimens from 64 children were collected, and clinical performance of mNGS pathogen detection capability when using BALF specimens from pediatric pulmonary tuberculosis patients was analyzed and compared with the detection performance of the CMTs method. Lung microecology was also analyzed using mNGS data, revealing the etiology of tuberculosis from another perspective, providing a basis for better understanding the pathogenesis of PTB in children, developing host-specific targeted therapies, and improving patient prognosis (Li et al., 2024; Roquilly et al., 2019).

*MTBC* consists mainly of *Mycobacterium tuberculosis*, *Mycobacterium bovis*, *Mycobacterium africanum*, *Mycobacterium canettii*, and other subtypes, which are capable of causing tuberculosis in humans (Gagneux, 2018). The principles of treatment are similar for the different subtypes in both adults and children, relying primarily on first-line antituberculosis drugs (e.g., isoniazid, rifampicin, ethambutol, and pyrazinamide) (Gagneux, 2018; Paleiron et al., 2019). As children have the highest TB morbidity and mortality, early intervention to detect latent infection and active disease in pediatric tuberculosis is preferable, which can significantly reduce drug side effects (Dodd et al., 2014; Global Tuberculosis Report, 2023). The current diagnostic abilities for the detection of pediatric PTB are suboptimal. Multiple factors contribute to the under-diagnosis of intrathoracic PTB in children, namely the absence of pathognomonic features of the disease, low bacillary loads in respiratory specimens, challenges in sample collection, and inadequate access to diagnostic tools in high-burden settings. Compared with the NTB patients, the pediatric patients with PTB had no specific clinical features. The utility of clinical symptoms for screening TB is hard to characterize, with rather low sensitivity and unsatisfactory specificity (Reuter et al., 2019). It is difficult to accurately discriminate PTB from community-acquired pneumonia (CAP) in the early stages of the diagnostic process by traditional methods, particularly in pediatric patients (Dheda et al., 2020; Grossman et al., 2014; Rueda et al., 2022). Recently, the use of mNGS for the direct detection of mycobacteria in clinical specimens has received considerable attention because of its advantage in shortened turnaround time and unbiased pathogen detection for diagnosis (Gu et al., 2019). When we comprehensively analyzed the TB diagnostic performance of the methods, we found that the sensitivity of the mNGS method for BALF samples of all childhood PTB cases was 46.51%, which was lower than that of TB-PCR (55.00%) and Xpert (55.00%), but the diagnostic value was much higher than that of the AFB and routine culture tests. Further combined analysis of mNGS and TB-PCR revealed the highest diagnostic rate (62.79%) for detecting tuberculosis in children (AUC=0.8140, *P*<0.0001), suggesting that combined mNGS and TB-PCR may improve the diagnostic strategy for tuberculosis diagnosis in children, which is in line with other studies (Shi et al., 2020; Zhou et al., 2019). Moreover, mNGS can also determine the species of NTB patients, which is difficult for culture-based methods because some species of NTB are difficult to grow during culture. Therefore, the results of mNGS were useful for providing appropriate and targeted treatment to pediatric patients in the early stage, supporting the clinical decision of treatment and helping to rule out infection.

The human respiratory tract is home to niche-specific communities of microorganisms that may serve as a gatekeeper, preventing respiratory pathogens from colonizing the respiratory system. In addition, the respiratory microbiota is thought to play a role in the maturation and maintenance of respiratory physiology and immunity, helping to maintain homeostasis in the respiratory system (Man et al., 2017). Although sputum samples have been used extensively as indicators of lung microbiota to study respiratory diseases, the samples are inevitably contaminated by the pharyngeal microbiota during the collection process (Molyneaux et al., 2013; Rogers et al., 2013; Zhao et al., 2012). Some previous studies have demonstrated that pharyngeal contamination has little effect on the microbiota harvested by the bronchoscopy (Bassis et al., 2015; Dickson et al., 2014; Natalini et al., 2023). We therefore chose to use BALF collected by bronchoscopy to investigate the lung microbiota in pediatric participants. Studying the lung microbiota of infected individuals in children is necessary to determine the relationship between microbial changes and respiratory disease. We analyzed the pediatric lung microbiome using BALF-mNGS data, including diversity, species composition, and high-frequency species. No significant differences were found in a diversity within the pediatric PTB and NTB groups. The lack of differences is mainly due to the fact that most pediatric patients in our study were exposed to antibiotics prior to mNGS, which may induce changes in the pathogenic infection. It also may have been influenced by the timing of BALF sampling. Collection time varied depending on clinical diagnostic needs and was not consistent in all patients over the course of the disease. Additionally, this may indirectly reflect a possible convergence of pulmonary microecological changes in pediatric PTB and pneumonia patients due to environmental and medication management, leading to decreased overall diversity with pathogenic microorganisms occupying the main ecological niche (Dai et al., 2018). Airway microbiotas were reported associated with an increased (risk microbiota) or decreased (resilience microbiota) incidence and severity of acute respiratory infection in children (Hasegawa and Camargo, 2015). In the LfFSe analysis, we found that in pediatric PTB and mNGS-positive-TB individuals, *Aquirufa antheringensis*, *Flavobacterium ammoniigenes*, *Aquirufa lenticrescens* and *Candidatus Planktophila sulfonica*, which are uncommon microorganisms in the body are increased in abundance, they are usually found in the environment or in the water column, and their direct association with the organism is less frequently reported (Neuenschwander et al., 2018; Pitt et al., 2022, 2019; Watanabe et al., 2022). The presence of these characteristic bacteria may suggest a role for environmental factors and immune status in the development of pediatric tuberculosis, where *M. tuberculosis* infection alters the pulmonary microenvironment, which may restrain certain microbes but induce growth for some other bacteria (Li et al., 2024). Within the pediatric pneumonia patient-NTB group, LfFSe analysis enriched for more pathogenic bacteria such as *Enterobacter quasiroggenkampii*, *Pseudomonas putida* and *Moraxella catarrhalis*, which are usually reported to be associated with the immunosuppressive organismal state (Cortazzo et al., 2023; Shaikh et al., 2023; von Bargen and Haas, 2009). Since immunodeficiency is recognized as a risk factor for pneumonia, it is reasonable to assume that these strains may serve as microbial markers indicative of pneumonia severity and prognosis. However, these species need further confirmation by other independent cohorts before being used as biomarkers.

There are several limitations in this study that should be noted. First, the number of individuals included was relatively small. We did not differentiate between training and test sets, which may lead to limited generalizability of the statistical findings. Further studies including a larger number of samples are required to confirm our findings. Multicenter studies are recommended to assess the value of mNGS in different pediatric populations. In addition, as oral samples were not collected for comparison with BALF in this study, contamination of BALF by oral flora could not be completely avoided. However, the abundance of oropharyngeal microbiota did not differ significantly between groups, i.e., potential oral contamination did not influence the results of this study. Finally, in this study, we focused only on bacteria and did not analyze viruses or fungi, which are also important for shaping the profile of the lung microbiota and should be considered in future studies.

In summary, this study provided a detailed characterization for the first time the potential advances in the etiological diagnosis of pediatric PTB using mNGS results of BALF, revealing information on pathogenicity and lung microbiome characteristics. For pediatric patients, it is important to consider the timing of early mNGS sampling and testing, while regular CMTs monitoring remains crucial. The combination of mNGS and CMTs can achieve high efficacy in pathogen testing, leading to faster diagnosis, improved medication regimens, and higher cure rates.

## Data Availability

The datasets presented in this study are available in an online repository. The names of the repository/repositories and accession number(s) can be found below: https://ngdc.cncb.ac.cn, PRJCA033530.

## Supplementary material

Supplementary Figure 1 Definition of test tuberculosis patients.

## References

[B1] Bassis C. M. Erb-Downward J. R. Dickson R. P. Freeman C. M. Schmidt T. M. Young V. B.. (2015). Analysis of the upper respiratory tract microbiotas as the source of the lung and gastric microbiotas in healthy individuals. mBio 6, e00037. doi: 10.1128/mBio.00037-15 25736890 PMC4358017

[B2] Chiu C. Y. Miller S. A. (2019). Clinical metagenomics. Nat. Rev. Genet. 20, 341–355. doi: 10.1038/s41576-019-0113-7 30918369 PMC6858796

[B3] Cortazzo V. Agosta M. Gaspari S. Vrenna G. Lucignano B. Onori M.. (2023). First case of VIM-1-like-producing pseudomonas putida bacteremia in an oncohematological pediatric patient in Italy. Antibiotics (Basel) 12 (6), 1033. doi: 10.3390/antibiotics12061033 37370352 PMC10295762

[B4] Dai W. Wang H. Zhou Q. Feng X. Lu Z. Li D.. (2018). The concordance between upper and lower respiratory microbiota in children with Mycoplasma pneumoniae pneumonia. Emerg. Microbes Infect. 7, 92. doi: 10.1038/s41426-018-0097-y 29789582 PMC5964150

[B5] de Blic J. Midulla F. Barbato A. Clement A. Dab I. Eber E.. (2000). Bronchoalveolar lavage in children. ERS Task Force on bronchoalveolar lavage in children. European Respiratory Society. Eur. Respir. J. 15, 217–231. doi: 10.1183/09031936.00.15121700 10678650

[B6] Dheda K. Makambwa E. Esmail A. (2020). The great masquerader: tuberculosis presenting as community-acquired pneumonia. Semin. Respir. Crit. Care Med. 41, 592–604. doi: 10.1055/s-0040-1710583 32564347

[B7] Dickson R. P. Martinez F. J. Huffnagle G. B. (2014). The role of the microbiome in exacerbations of chronic lung diseases. Lancet 384, 691–702. doi: 10.1016/s0140-6736(14)61136-3 25152271 PMC4166502

[B8] Dodd P. J. Gardiner E. Coghlan R. Seddon J. A. (2014). Burden of childhood tuberculosis in 22 high-burden countries: a mathematical modelling study. Lancet Glob Health 2, e453–e459. doi: 10.1016/s2214-109x(14)70245-1 25103518

[B9] Gagneux S. (2018). Ecology and evolution of Mycobacterium tuberculosis. Nat. Rev. Microbiol. 16, 202–213. doi: 10.1038/nrmicro.2018.8 29456241

[B10] Global tuberculosis report. (2023). Available online at: https://www.who.int/teams/global-tuberculosis-programme/tb-reports (Accessed September 06, 2024).

[B11] Grossman R. F. Hsueh P. R. Gillespie S. H. Blasi F. (2014). Community-acquired pneumonia and tuberculosis: differential diagnosis and the use of fluoroquinolones. Int. J. Infect. Dis. 18, 14–21. doi: 10.1016/j.ijid.2013.09.013 24211230

[B12] Gu W. Miller S. Chiu C. Y. (2019). Clinical metagenomic next-generation sequencing for pathogen detection. Annu. Rev. Pathol. 14, 319–338. doi: 10.1146/annurev-pathmechdis-012418-012751 30355154 PMC6345613

[B13] Haas M. K. Belknap R. W. (2018). Updates in the treatment of active and latent tuberculosis. Semin. Respir. Crit. Care Med. 39, 297–309. doi: 10.1055/s-0038-1660863 30071545

[B14] Han D. Li Z. Li R. Tan P. Zhang R. Li J. (2019). mNGS in clinical microbiology laboratories: on the road to maturity. Crit. Rev. Microbiol. 45, 668–685. doi: 10.1080/1040841x.2019.1681933 31691607

[B15] Hasegawa K. Camargo C. A. Jr. (2015). Airway microbiota and acute respiratory infection in children. Expert Rev. Clin. Immunol. 11, 789–792. doi: 10.1586/1744666x.2015.1045417 25961472 PMC4828966

[B16] He Y. Lyon C. J. Nguyen D. T. Liu C. Sha W. Graviss E. A.. (2021). Serum-based diagnosis of pediatric tuberculosis by assay of mycobacterium tuberculosis factors: a retrospective cohort study. J. Clin. Microbiol. 59 (2), e01756-20. doi: 10.1128/jcm.01756-20 33239373 PMC8111146

[B17] Hogan C. A. Yang S. Garner O. B. Green D. A. Gomez C. A. Dien Bard J.. (2021). Clinical impact of metagenomic next-generation sequencing of plasma cell-free DNA for the diagnosis of infectious diseases: A multicenter retrospective cohort study. Clin. Infect. Dis. 72, 239–245. doi: 10.1093/cid/ciaa035 31942944

[B18] Hu Y. Kang Y. Liu X. Cheng M. Dong J. Sun L.. (2020). Distinct lung microbial community states in patients with pulmonary tuberculosis. Sci. China Life Sci. 63, 1522–1533. doi: 10.1007/s11427-019-1614-0 32303963

[B19] Lee R. A. Al Dhaheri F. Pollock N. R. Sharma T. S. (2020). Assessment of the clinical utility of plasma metagenomic next-generation sequencing in a pediatric hospital population. J. Clin. Microbiol. 58 (7), e00419-20. doi: 10.1128/jcm.00419-20 32376666 PMC7315020

[B20] Lewinsohn D. M. Leonard M. K. LoBue P. A. Cohn D. L. Daley C. L. Desmond E.. (2017). Official american thoracic society/infectious diseases society of america/centers for disease control and prevention clinical practice guidelines: diagnosis of tuberculosis in adults and children. Clin. Infect. Dis. 64, 111–115. doi: 10.1093/cid/ciw778 28052967 PMC5504475

[B21] Li R. Li J. Zhou X. (2024). Lung microbiome: new insights into the pathogenesis of respiratory diseases. Signal Transduct Target Ther. 9, 19. doi: 10.1038/s41392-023-01722-y 38228603 PMC10791971

[B22] Man W. H. de Steenhuijsen Piters W. A. Bogaert D. (2017). The microbiota of the respiratory tract: gatekeeper to respiratory health. Nat. Rev. Microbiol. 15, 259–270. doi: 10.1038/nrmicro.2017.14 28316330 PMC7097736

[B23] Mandal N. Anand P. K. Gautam S. Das S. Hussain T. (2017). Diagnosis and treatment of paediatric tuberculosis: An insight review. Crit. Rev. Microbiol. 43, 466–480. doi: 10.1080/1040841x.2016.1262813 28502224

[B24] Miao Q. Ma Y. Wang Q. Pan J. Zhang Y. Jin W.. (2018). Microbiological diagnostic performance of metagenomic next-generation sequencing when applied to clinical practice. Clin. Infect. Dis. 67, S231–s240. doi: 10.1093/cid/ciy693 30423048

[B25] Miller S. Chiu C. (2021). The role of metagenomics and next-generation sequencing in infectious disease diagnosis. Clin. Chem. 68, 115–124. doi: 10.1093/clinchem/hvab173 34969106

[B26] Molyneaux P. L. Mallia P. Cox M. J. Footitt J. Willis-Owen S. A. Homola D.. (2013). Outgrowth of the bacterial airway microbiome after rhinovirus exacerbation of chronic obstructive pulmonary disease. Am. J. Respir. Crit. Care Med. 188, 1224–1231. doi: 10.1164/rccm.201302-0341OC 23992479 PMC3863728

[B27] Natalini J. G. Singh S. Segal L. N. (2023). The dynamic lung microbiome in health and disease. Nat. Rev. Microbiol. 21, 222–235. doi: 10.1038/s41579-022-00821-x 36385637 PMC9668228

[B28] Neuenschwander S. M. Ghai R. Pernthaler J. Salcher M. M. (2018). Microdiversification in genome-streamlined ubiquitous freshwater Actinobacteria. Isme J. 12, 185–198. doi: 10.1038/ismej.2017.156 29027997 PMC5739012

[B29] Paleiron N. Soler C. Hassan M. O. Andriamanantena D. Vong R. Pourcel C.. (2019). First description of Mycobacterium tuberculosis and M. canettii concomitant infection: report of two cases. Int. J. Tuberc Lung Dis. 23, 232–235. doi: 10.5588/ijtld.18.0261 30688210

[B30] Pitt A. Koll U. Schmidt J. Neumann-Schaal M. Wolf J. Krausz S.. (2022). Aquirufa lenticrescens sp. nov. and Aquirufa aurantiipilula sp. nov.: two new species of a lineage of widespread freshwater bacteria. Arch. Microbiol. 204, 356. doi: 10.1007/s00203-022-02950-6 35654990 PMC9163014

[B31] Pitt A. Schmidt J. Koll U. Hahn M. W. (2019). Aquirufa antheringensis gen. nov., sp. nov. and Aquirufa nivalisilvae sp. nov., representing a new genus of widespread freshwater bacteria. Int. J. Syst. Evol. Microbiol. 69, 2739–2749. doi: 10.1099/ijsem.0.003554 31259682

[B32] Prasad R. Singh A. Gupta N. (2019). Adverse drug reactions in tuberculosis and management. Indian J. Tuberc 66, 520–532. doi: 10.1016/j.ijtb.2019.11.005 31813444

[B33] Reuter A. Hughes J. Furin J. (2019). Challenges and controversies in childhood tuberculosis. Lancet 394, 967–978. doi: 10.1016/s0140-6736(19)32045-8 31526740

[B34] Rogers G. B. van der Gast C. J. Cuthbertson L. Thomson S. K. Bruce K. D. Martin M. L.. (2013). Clinical measures of disease in adult non-CF bronchiectasis correlate with airway microbiota composition. Thorax 68, 731–737. doi: 10.1136/thoraxjnl-2012-203105 23564400

[B35] Roquilly A. Torres A. Villadangos J. A. Netea M. G. Dickson R. Becher B.. (2019). Pathophysiological role of respiratory dysbiosis in hospital-acquired pneumonia. Lancet Respir. Med. 7, 710–720. doi: 10.1016/s2213-2600(19)30140-7 31182406

[B36] Rueda Z. V. Aguilar Y. Maya M. A. López L. Restrepo A. Garcés C.. (2022). Etiology and the challenge of diagnostic testing of community-acquired pneumonia in children and adolescents. BMC Pediatr. 22, 169. doi: 10.1186/s12887-022-03235-z 35361166 PMC8968093

[B37] Sala C. Benjak A. Goletti D. Banu S. Mazza-Stadler J. Jaton K.. (2020). Multicenter analysis of sputum microbiota in tuberculosis patients. PloS One 15, e0240250. doi: 10.1371/journal.pone.0240250 33044973 PMC7549818

[B38] Shaikh N. Hoberman A. Shope T. R. Jeong J. H. Kurs-Lasky M. Martin J. M.. (2023). Identifying children likely to benefit from antibiotics for acute sinusitis: A randomized clinical trial. Jama 330, 349–358. doi: 10.1001/jama.2023.10854 37490085 PMC10370259

[B39] Shi C. L. Han P. Tang P. J. Chen M. M. Ye Z. J. Wu M. Y.. (2020). Clinical metagenomic sequencing for diagnosis of pulmonary tuberculosis. J. Infect. 81, 567–574. doi: 10.1016/j.jinf.2020.08.004 32768450

[B40] Simner P. J. Miller S. Carroll K. C. (2018). Understanding the promises and hurdles of metagenomic next-generation sequencing as a diagnostic tool for infectious diseases. Clin. Infect. Dis. 66, 778–788. doi: 10.1093/cid/cix881 29040428 PMC7108102

[B41] Tao Y. Yan H. Liu Y. Zhang F. Luo L. Zhou Y.. (2022). Diagnostic performance of metagenomic next-generation sequencing in pediatric patients: A retrospective study in a large children’s medical center. Clin. Chem. 68, 1031–1041. doi: 10.1093/clinchem/hvac067 35704075

[B42] Thomas T. A. (2017). Tuberculosis in children. Pediatr. Clin. North Am. 64, 893–909. doi: 10.1016/j.pcl.2017.03.010 28734517 PMC5555046

[B43] Ticlla M. R. Hella J. Hiza H. Sasamalo M. Mhimbira F. Rutaihwa L. K.. (2021). The sputum microbiome in pulmonary tuberculosis and its association with disease manifestations: A cross-sectional study. Front. Microbiol. 12. doi: 10.3389/fmicb.2021.633396 PMC8417804 34489876

[B44] Turkova A. Wills G. H. Wobudeya E. Chabala C. Palmer M. Kinikar A.. (2022). Shorter treatment for nonsevere tuberculosis in african and Indian children. N Engl. J. Med. 386, 911–922. doi: 10.1056/NEJMoa2104535 35263517 PMC7612496

[B45] Valdez-Palomares F. Muñoz Torrico M. Palacios-González B. Soberón X. Silva-Herzog E. (2021). Altered microbial composition of drug-sensitive and drug-resistant TB patients compared with healthy volunteers. Microorganisms 9 (8), 1762. doi: 10.3390/microorganisms9081762 34442841 PMC8398572

[B46] von Bargen K. Haas A. (2009). Molecular and infection biology of the horse pathogen Rhodococcus equi. FEMS Microbiol. Rev. 33, 870–891. doi: 10.1111/j.1574-6976.2009.00181.x 19453748

[B47] Watanabe K. Kitamura T. Ogata Y. Shindo C. Suda W. (2022). Flavobacterium ammonificans sp. nov. and Flavobacterium ammoniigenes sp. nov., ammonifying bacteria isolated from surface river water. Int. J. Syst. Evol. Microbiol. 72. doi: 10.1099/ijsem.0.005307 35344478

[B48] WHO Guidelines Approved by the Guidelines Review Committee (2022). WHO consolidated guidelines on tuberculosis: Module 3: Diagnosis – Tests for tuberculosis infection (Geneva: World Health Organization). 36441853

[B49] Zhao J. Schloss P. D. Kalikin L. M. Carmody L. A. Foster B. K. Petrosino J. F.. (2012). Decade-long bacterial community dynamics in cystic fibrosis airways. Proc. Natl. Acad. Sci. U.S.A. 109, 5809–5814. doi: 10.1073/pnas.1120577109 22451929 PMC3326496

[B50] Zhou X. Wu H. Ruan Q. Jiang N. Chen X. Shen Y.. (2019). Clinical Evaluation of Diagnosis Efficacy of Active Mycobacterium tuberculosis Complex Infection via Metagenomic Next-Generation Sequencing of Direct Clinical Samples. Front. Cell Infect. Microbiol. 9. doi: 10.3389/fcimb.2019.00351 PMC6813183 31681628

