# Supplementary figures and images for: Diagnosis and insight into the unique lung microbiota of pediatric pulmonary tuberculosis patients by bronchoalveolar lavage using metagenomic next-generation sequencing
Source: Front Cell Infect Microbiol. 2024 Dec 19;14:1492881. doi: 10.3389/fcimb.2024.1492881 (PMC11693512; doi:10.3389/fcimb.2024.1492881)

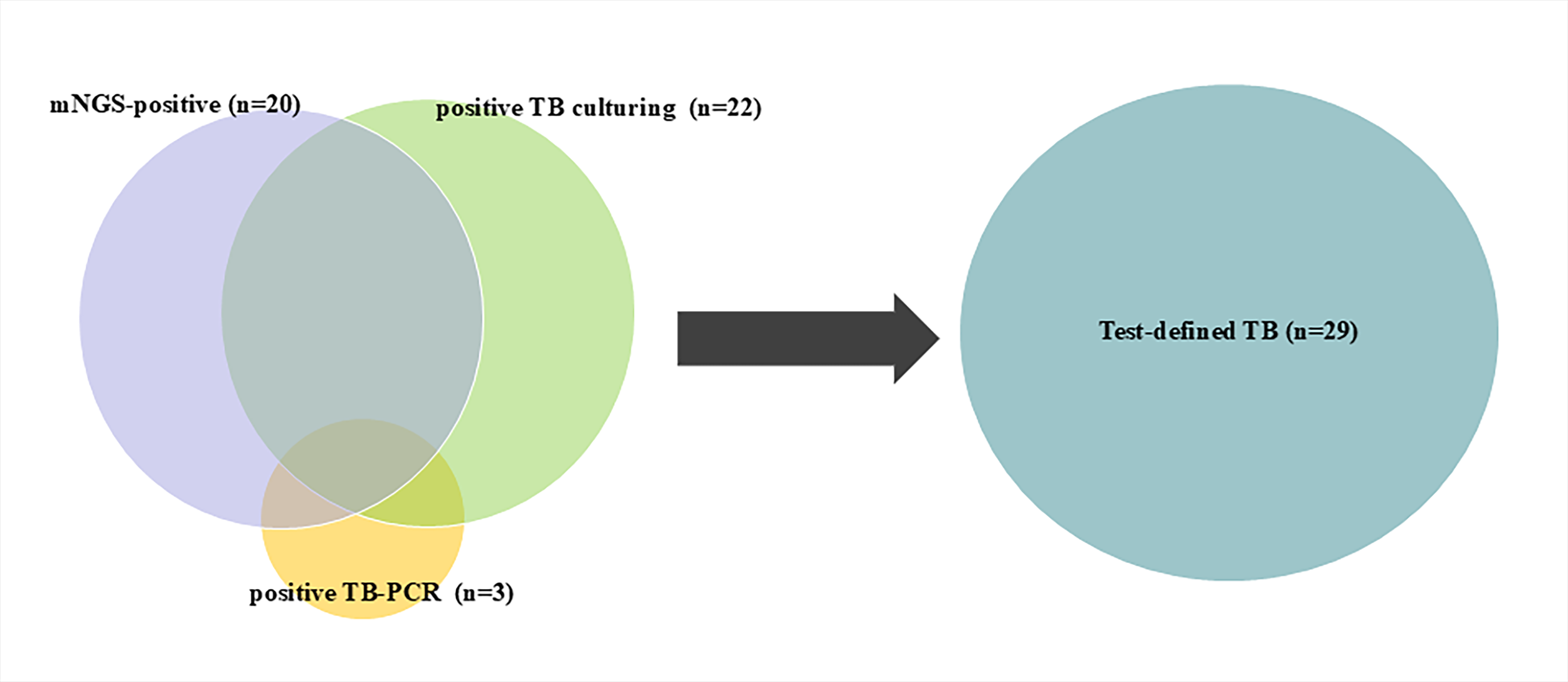

Supplement: Supplementary Figure 1 — Definition of test tuberculosis patients. [file Image1.tif]
